# Lyme Neuroborreliosis Presenting As Diplopia and Syndrome of Inappropriate Antidiuretic Hormone Secretion

**DOI:** 10.7759/cureus.105850

**Published:** 2026-03-25

**Authors:** Hugh Johnson, Hashim Abbas, Thu-Thao Ly, Darrell McBride

**Affiliations:** 1 Medical Education, Geisinger Commonwealth School of Medicine, Scranton, USA; 2 Nephrology, Geisinger Medical Center, Danville, USA; 3 Neuroradiology, Geisinger Medical Center, Danville, USA; 4 Infectious Diseases, Geisinger Medical Center, Danville, USA

**Keywords:** demyelination, hyponatremia, lyme disease, lyme neuroborreliosis, syndrome of inappropriate antidiuretic hormone secretion (siadh)

## Abstract

Lyme neuroborreliosis represents the neurologic manifestations of disseminated Lyme disease, typically presenting as meningitis, cranial neuropathy, or radiculoneuropathy. Although syndrome of inappropriate antidiuretic hormone secretion (SIADH) is a recognized complication of central nervous system infections, it is not commonly associated with Lyme neuroborreliosis, and only a few linked cases have been reported in the literature.

This case describes a male in his 50s who presented to the emergency department with severe headache, diplopia with inability to abduct the right eye, back pain, and hyponatremia secondary to SIADH. Initial neuroimaging revealed optic neuritis, and CSF analysis revealed lymphocytic pleocytosis, elevated protein, and an increased total nucleated cell count. Oligoclonal bands were also detected in the CSF. While multiple sclerosis was initially clinically suspected, Lyme disease screening via enzyme-linked immunosorbent assay (ELISA) was performed because of the patient’s location in the Northeastern United States and returned positive. Subsequent confirmatory Western blot analysis was positive for IgG and negative for IgM. The patient was treated with IV ceftriaxone followed by a 21-day course of doxycycline, after which his neurologic symptoms, headache, and hyponatremia resolved.

## Introduction

Lyme disease is a multisystem infectious disease caused by the spirochete *Borrelia burgdorferi*, which is transmitted by the *Ixodes scapularis* tick. It is the most common vector-borne disease in the United States, particularly in the Northeast. Incidence has increased over past decades, with 89,468 cases reported in the United States in 2023 [1]. The epidemiologic significance of Lyme disease is further supported by historic underreporting of Lyme cases [2]. Of the systems affected, neurologic manifestations, dubbed Lyme neuroborreliosis, occur in about 12% of patients with Lyme disease [3]. These neurologic manifestations typically include, but are not limited to, cranial neuropathies (especially facial palsy), lymphocytic meningitis, and painful meningoradiculitis [4]. The range of clinical presentations of Lyme neuroborreliosis poses the potential for misdiagnosis as other neurologic pathologies, and correct detection is essential for proper treatment to prevent disease progression.

Hyponatremia, defined as a serum sodium level below 135 mmol/L, is a frequent medical occurrence, affecting up to 35% of hospitalized patients [5]. One notable cause is syndrome of inappropriate antidiuretic hormone secretion (SIADH), characterized by euvolemic hypotonic hyponatremia resulting from the inappropriate secretion of antidiuretic hormone in the absence of hypertonicity or decreased arterial blood volume [5]. There are several known causes of SIADH, such as malignancy, medications, pulmonary infections, or CNS trauma/infection. CNS infection is a recognized cause of SIADH, particularly with microbes such as Mycobacterium tuberculosis, and recent studies have attributed 77.7% of meningitis-associated hyponatremia to SIADH [6]. Lyme neuroborreliosis, however, is not typically associated with this syndrome, and only a few cases have been reported in the literature [7-13]. Identification and treatment of these causal associations are essential in the overall management of SIADH [5].

We present a case of a male in his 50s with chronic back pain, severe headache, diplopia, and hyponatremia secondary to SIADH.

## Case presentation

A male in his late 50s presented to our ED with a four-week history of progressive bifrontal headaches and monocular diplopia worsened when looking to the right. The headaches had gradually increased from mild to 10/10 in severity and were described as pressure-like and bifrontal in location. This pain was non-radiating, had no aggravating factors, and was not alleviated by acetaminophen or ibuprofen. He had recently been admitted to and discharged from another hospital 3 days earlier for these symptoms, where he was treated for hyponatremia with urea tablets and fluid restriction. He was also given sumatriptan for his headaches, which he reported as ineffective. The patient was then seen by his primary care physician and sent to our ED for further evaluation of possible CNS involvement. Upon review of systems at the time of examination, the patient denied fever, photophobia, phonophobia, nausea, or vomiting.

Past medical history included chronic back pain secondary to T8-T12 vertebral compression fractures due to a past workplace accident, peripheral neuropathy, a 44-pack-year tobacco history, and alcohol use. He reported consuming one to two beers daily for several years. Medications before admission included gabapentin, baclofen, famotidine, urea tablets, and prochlorperazine for nausea experienced during his recent hospitalization.

Upon examination, vital signs were within normal limits. The patient was afebrile, and cardiovascular, pulmonary, and abdominal examinations were unremarkable. He did not have neck stiffness or tenderness, and no lymphadenopathy was noted in the neck or axilla. On neurologic examination, the patient was noted to have limited ability to abduct his right eye. He described right-sided predominant blurred vision and diplopia that worsened when asked to look to the right, consistent with possible cranial nerve VI palsy. Other than right eye blurriness, vision was intact in all quadrants in both eyes to finger counting on examination, though Snellen testing was not performed on the initial examination. Cranial nerves II-XII were otherwise intact on neurologic examination, and no sensory, motor, or mental status deficits were noted. Reflexes and muscle strength were normal.

**Figure 1:**
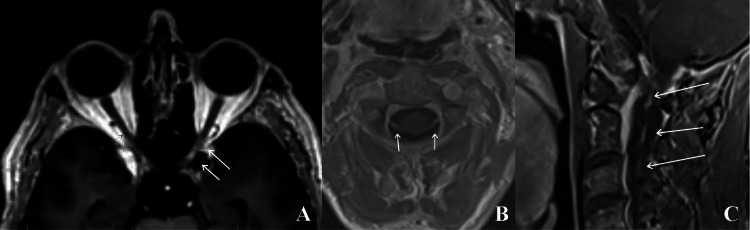
Contrast-enhanced MRI of the brain and cervical spine nerve roots. (A) Axial T1-weighted MRI with contrast. Solid white arrows show abnormal signal changes in the left optic nerve, indicating optic neuritis. This finding is interesting considering that the patient did not demonstrate associated symptoms such as monocular left-eye vision loss or pain with eye movement. (B) Axial T1-weighted MRI with contrast. Arrows show dorsal cervical nerve root enhancement. (C) Sagittal T1-weighted MRI with contrast. Arrows show dorsal cervical nerve root enhancement. These inflammatory findings in the nerve roots are unlikely in central inflammatory pathologies such as multiple sclerosis. Imaging parameters: A: Spin-echo T1-weighted MRI with contrast; image resolution: 1.0 mm × 1.0 mm; slice thickness: 1.0 mm. B: Gradient-recalled T1-weighted MRI with contrast; matrix size: 256 × 248; slice thickness: 2.0 mm. C: Spin-echo T1-weighted MRI post-contrast with fat suppression; matrix size: 288 × 202; slice thickness: 3.0 mm.

The initial impression of this patient’s vision changes and severe headache raised suspicion for an intracranial etiology, prompting computed tomography angiography (CTA) of the head and neck, which revealed no acute findings. MRI of the cervical and thoracic spine and brain with contrast was then performed. Brain MRI interestingly revealed left optic nerve enhancement (Figure 1A), despite his vision symptoms pertaining to the right eye. No evidence of acute ischemic infarction or lesion was noted on brain imaging.

**Figure 2:**
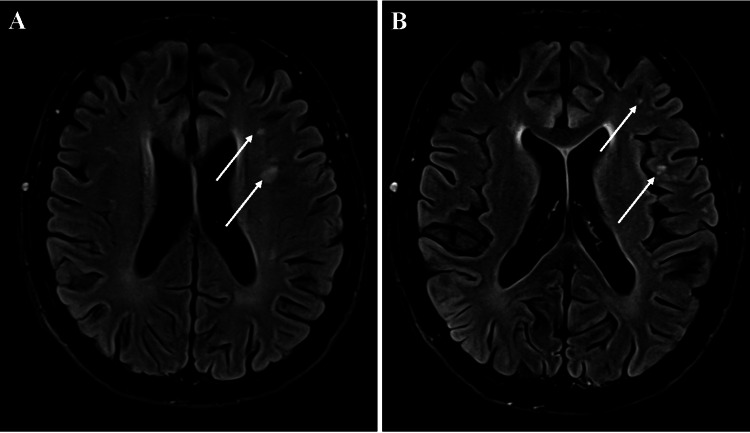
Axial T1-weighted FLAIR MRI of the brain at the level of the lateral ventricles. (A) Superior view. (B) Inferior view. Nonspecific, patchy white matter lesions are highlighted by white arrows and may be secondary to inflammation, infection, trauma, or other causes, including demyelination. Imaging parameters: Spin-echo T1-weighted FLAIR MRI with contrast; matrix size: 256 × 162; slice thickness: 5.0 mm. FLAIR: Fluid-attenuated inversion recovery.

On subsequent review of the T1-weighted MRI images, subtle enhancement can be appreciated along the dorsal cervical nerve roots, most notably in the upper cervical spine (Figures 1B-1C). Axial fluid-attenuated inversion recovery (FLAIR) images also reflect nonspecific, patchy white matter lesions within the cortical and periventricular white matter of the brain (Figures 2A-2B).

Laboratory studies in the ED revealed hyponatremia with a serum sodium of 125 mEq/L, chloride of 90 mEq/L, and serum osmolality of 261 mOsm/kg (Table 1). Initial chest X-ray in the ED revealed small right and left pleural effusions and a small lateral right mid-lung opacity, for which follow-up CT imaging was recommended for better resolution. Chest CT was then performed, revealing a tree-in-bud nodular opacity in the right upper lobe as well as debris within the trachea. These results were interpreted as possible infectious or inflammatory processes, such as aspiration pneumonia.

**Table 1:** Metabolic panel and blood count testing on hospital day 1. Na: Sodium; K: Potassium; Cl: Chloride; BUN: Blood urea nitrogen; Cr: Creatinine; eGFR: Estimated glomerular filtration rate; Hgb: Hemoglobin; Hct: Hematocrit; PLT: Platelet count; MCV: Mean corpuscular volume; MCH: Mean corpuscular hemoglobin; RDW: Red cell distribution width; MPV: Mean platelet volume; mmol/L: Millimoles per liter; mg/dL: Milligrams per deciliter; mL/min: Milliliters per minute; mOsm/kg: Milliosmoles per kilogram; K/µL: Thousands per microliter; M/µL: Millions per microliter; g/dL: Grams per deciliter; fL: Femtoliters; pg: Picograms.

Lab Test	Recorded Value	Reference Value
Na (mmol/L)	125	135-146
K (mmol/L)	4.3	3.5-5.1
Cl (mmol/L)	90	98-107
BUN (mg/dL)	17	6-20
Cr (mg/dL)	0.7	0.6-1.2
eGFR (mL/min)	>90	>60
Anion gap	8	7-15
Glucose (mg/dL)	91	70-120
Ca (mg/dL)	9.6	8.4-10.2
Urine sodium (mmol/L)	75	40-220
Urine osmolality (mOsm/kg)	525	50-1200
Serum osmolality (mOsm/kg)	261	278-305
WBC (K/µL)	6.86	4.0-10.8
RBC (M/µL)	5.1	4.28-5.64
Hgb (g/dL)	16.4	13.0-16.9
Hct (%)	46.6	39.1-49.5
PLT (K/µL)	302	140-400
MCV (fL)	91.4	82.0-99.5
MCH (pg)	32.2	27.0-34.0
RDW (%)	12	11.5-15.5
MPV (fL)	9.9	6.6-11.1

The patient was then admitted for his electrolyte abnormalities and for continued workup of his symptoms. Further investigation of his hyponatremia revealed a random urine sodium of 75 mmol/L and urine osmolality of 525 mOsm/kg, indicating SIADH. Lumbar puncture was performed, and testing revealed CSF protein of 108 mg/dL, glucose of 52 mg/dL, and 167/µL total nucleated cells (Table 2). The CSF infectious meningitis PCR panel was negative for potential organisms. CSF testing additionally revealed the presence of oligoclonal bands, CSF IgG of 7.9 mg/dL, and a CSF IgG index of 0.58. Serum antibody testing for myelin oligodendrocyte glycoprotein antibody-associated disease (MOGAD) and neuromyelitis optica (NMO) was also performed, both of which were negative.

**Table 2:** CSF analysis on hospital day 1. IgG: Immunoglobulin G; cells/µL: Cells per microliter; mg/dL: Milligrams per deciliter; mg/24 h: Milligrams per 24 hours.

Lab Test	Recorded Value	Reference Range
Total nucleated cells (cells/µL)	167	<5
RBC (cells/µL)	0	<5
Protein (mg/dL)	108	15-45
Glucose (mg/dL)	52	45-70
Neutrophils (%)	0.3	0.0-6.0
Lymphocytes (%)	84.7	40.0-80.0
Monocytes (%)	13.7	15.0-45.0
Basophils (%)	0.7	0
Plasma cells (%)	0.7	0.0-1.0
Color	Clear	Clear
Oligoclonal bands (IgG)	Present	Absent
CSF IgG (mg/dL)	7.9	0.8-7.7
CSF IgG synthesis rate (mg/24 h)	6.8	-9.9 to -3.3
CSF IgG index	0.58	<0.70
Albumin (mg/dL)	75.3	11-48

After initial imaging studies ruled out acute intracranial ischemia, mass effect, or hemorrhage, early clinical suspicion consisted of three differential diagnoses (Table 3). First, optic neuritis and suspected cranial nerve VI palsy, alongside IgG oligoclonal bands in the CSF, raised suspicion for multiple sclerosis. Second, hyponatremia, recent 10-lb weight loss, smoking history, and an inconclusive chest X-ray could indicate SIADH in the setting of lung malignancy. Finally, infectious meningitis could explain this patient’s headache, cranial nerve palsy, and CSF findings, including elevated protein and total nucleated cell count.

**Table 3:** Summary of differential diagnoses. SIADH: Syndrome of inappropriate antidiuretic hormone secretion.

Diagnosis	Findings Supporting	Findings Opposing
Lyme neuroborreliosis	Positive serum two-tier testing; peripheral and central nervous system inflammation on MRI; CSF pleocytosis and elevated protein; endemic location	Lack of classic Lyme presentation (bull’s-eye rash, flu-like prodrome)
Other infectious meningitis	Headache and SIADH; elevated CSF protein	Negative PCR testing for infectious organisms; lymphocytic CSF predominance
Multiple sclerosis	CSF oligoclonal bands; optic neuritis and diplopia; lymphocytic pleocytosis	Age and sex of the patient; peripheral nervous system involvement on MRI
Lung malignancy	Smoking history; recent weight loss; SIADH linked with small cell lung cancer; initially ambiguous chest X-ray findings	

Serum screening for Lyme disease based on the presentation in the Northeastern United States then returned and revealed a positive Lyme antibody ELISA screening result. Further Western blot confirmatory testing was positive for IgG and negative for IgM. This indicated Lyme disease in the late or disseminated stage rather than acute infection. Once informed about this finding, the patient could not recall any bull’s-eye rashes or flu-like symptoms within the last year, but he did recall possibly finding a tick on his skin several months earlier.

The patient’s symptoms of headache and inability to abduct his right eye persisted for two days after his initial admission, as well as back pain at the site of his lumbar puncture. Once Lyme meningitis was suspected, 2 g IV ceftriaxone every 24 hours was started, with an eventual plan to switch to doxycycline as per the infectious disease consult. IV ketorolac and a lidocaine patch somewhat improved his back pain, and his hyponatremia was treated with a 1 L daily fluid restriction, LiquaCel, and 15 g urea packets twice daily. His sodium level remained low despite these interventions during the three days following his admission.

As per the patient’s wishes, he was discharged on hospital day 4 with plans to continue doxycycline therapy 100 mg once daily for 21 days. Because his sodium had not returned to baseline at the time of discharge, he was sent home with 1 g sodium chloride tablets to take three times daily and a small 10 mg dose of furosemide to take until his sodium hopefully normalized. This decision was made because of issues with obtaining urea packets following his previous discharge.

**Figure 3:**
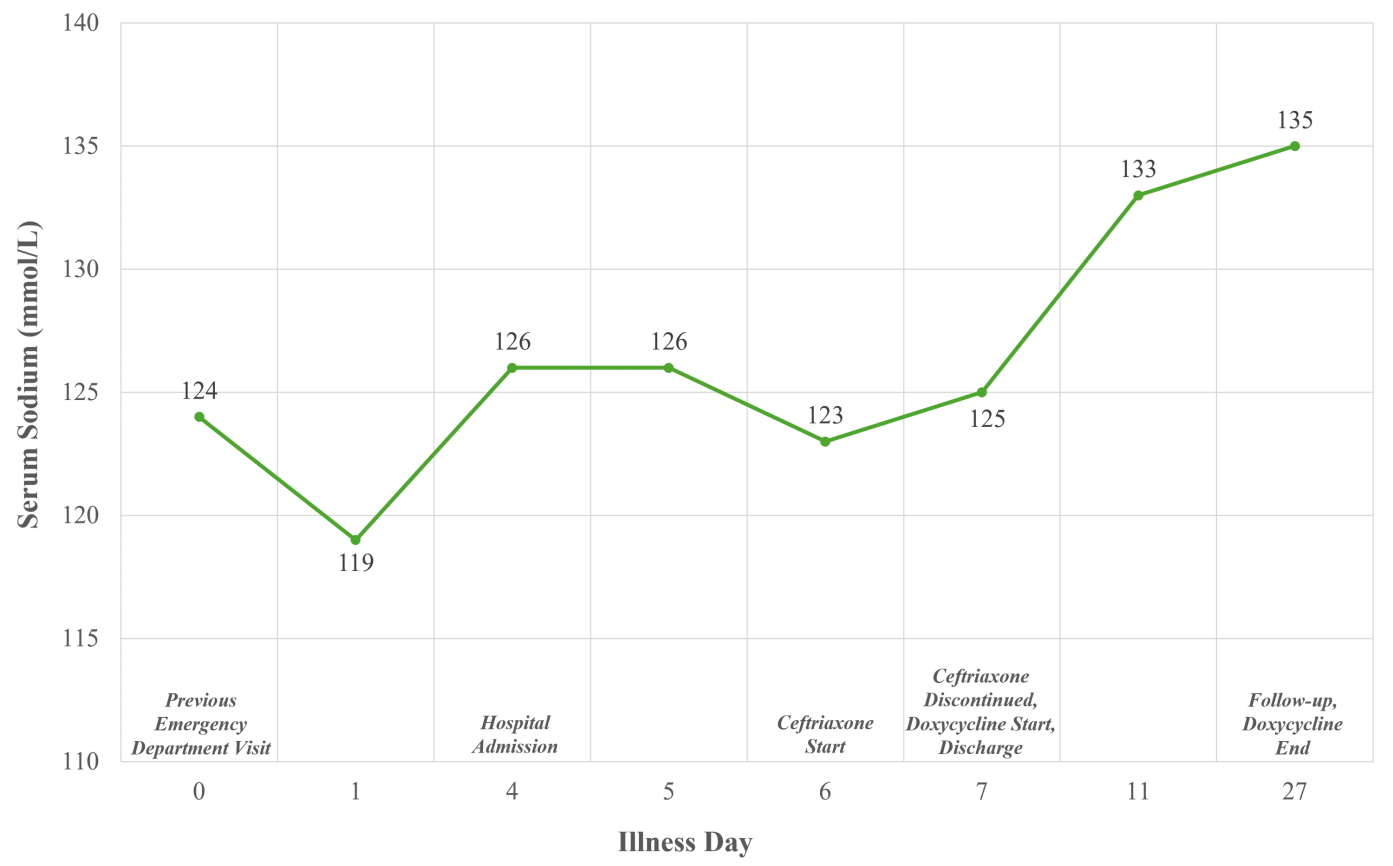
Timeline depicting illness course and serum sodium levels.

Follow-up laboratory work was done to monitor sodium levels. After 5 days of doxycycline treatment, the patient’s sodium had increased to 133 mmol/L, and 20 days after discharge, repeat laboratory tests revealed that the patient’s sodium had increased to 135 mmol/L (Figure 3). The patient’s headaches and visual symptoms resolved by 20 days after discharge, but his back pain persisted. These outcomes, coinciding with positive Lyme serum testing and antibiotic treatment, indicated Lyme neuroborreliosis as the probable etiology of this patient’s neurologic and SIADH symptoms. There was some consideration at this point for Lyme radiculitis as a source of the back pain, but the patient’s history of vertebral compression fractures and pain long preceding his new neurologic symptoms, which had otherwise resolved with antibiotics, made Bannwarth syndrome unlikely.

## Discussion

This case demonstrates the importance of considering Lyme disease as a potential etiology of SIADH in patients with hyponatremia and neurologic symptoms. While CNS infections are a recognized cause of SIADH, Lyme neuroborreliosis is not a typical association, with literature review revealing only a few documented cases of SIADH in Lyme disease [7-13].

Diagnostic approach

The identification of Lyme neuroborreliosis can be challenging in the absence of classic Lyme disease symptoms, such as erythema migrans rash or a flu-like prodrome [4]. Notably, a retrospective study indicated that 13% of North American patients diagnosed with Lyme disease did not present with the characteristic bull’s-eye rash [14] and, furthermore, Lyme meningitis can occasionally be the first sign of Lyme disease [15]. Lyme meningitis is categorized into early and late disseminated stages, with late-stage infection involving parenchymal changes that may be misinterpreted as multiple sclerosis [15]. Additionally, although rare, Lyme disease has been documented to affect the optic nerve, as observed in this patient [16]. In this case, the initial differential diagnosis was further complicated by the presence of CSF oligoclonal bands, which strengthened early suspicions for multiple sclerosis. This patient’s diagnostic course exemplified Lyme neuroborreliosis as a “great imitator” by mimicking other neurologic conditions.

Screening for Lyme disease therefore is especially important in cases that lack classic symptoms or mimic other diseases. As applicable to this case, screening is recommended in patients with acute neurologic syndromes such as meningitis, cranial neuropathies, or spinal inflammation in the setting of possible exposure to ticks infected with *Borrelia burgdorferi* [17]. The screening and diagnosis of Lyme disease are made with two-tiered serologic testing, in which positive ELISA screening is then confirmed with Western blot for IgG and IgM antibodies. This methodology remains common practice because of poor specificity in first-tier assays [3]. Guidelines for the confirmation of Lyme neuroborreliosis recommend detection of intrathecal antibodies to *B. burgdorferi* via determination of an antibody index (AI), which uses paired samples of both CSF and serum antibodies [18]. This methodology differentiates intrathecal antibody production from passive diffusion of antibodies from the serum through the blood-brain barrier, which occurs in approximately 0.5 to 1% of peripheral blood IgG antibodies [18]. While the AI method has high specificity and has been extensively studied and implemented in European populations, it has not yet been widely accepted in the United States. A lack of FDA-cleared tests for detection of CSF *B. burgdorferi* antibodies and few available laboratories offering AI testing in the United States make this method currently difficult and time intensive.

Other methods of testing, such as culturing for *Borrelia burgdorferi*, are not commonly used because of long culture times and low sensitivity. However, culturing may be considered in cases in which antibody assays are negative despite a clinical picture suspicious for Lyme [19]. Similarly, PCR testing also has low sensitivity, particularly in CSF, where PCR detected Borrelia in only 10-30% of patients with neuroborreliosis [19].

Imaging interpretation

Initial imaging in this case did not particularly suggest Lyme neuroborreliosis, but subsequent review of the MRI provides insight into features that could have helped guide the working diagnosis. MRI revealed subtle dorsal cranial nerve enhancement, especially in the upper cervical spine. This finding would have been impactful in the evaluation of the initial differential diagnoses, as multiple sclerosis demyelination typically is limited to the brain, optic tracts, and spinal cord rather than the peripheral nervous system. Furthermore, inflammatory neuritis findings on MRI are often seen in Lyme neuroborreliosis, in which the facial and oculomotor nerves are frequently affected [20]. The nonspecific white matter lesions seen in this patient also did not align with classic multiple sclerosis imaging findings, in which periventricular (“Dawson’s finger”), juxtacortical, and callosal FLAIR hyperintensities would be expected. While demyelination could not be ruled out, the MRI in this case could have been used more effectively in guiding the differential diagnosis.

Diagnostic reasoning

Other potential causes of this patient's SIADH included alternative causes of infective meningitis. However, the CSF meningitis PCR panel tested negative for common causative pathogens such as *Streptococcus pneumoniae* and *Neisseria meningitidis*, making other infectious organisms unlikely contributors. Although NSAID use has been associated with SIADH [5], this patient's hyponatremia resolved in parallel with Lyme diagnosis and treatment. Furthermore, the patient in this case continued to receive NSAIDs during and after his hospitalization without recurrence of hyponatremia. Malignancy was also initially considered as a cause of SIADH, given this patient's smoking history and inconclusive chest X-ray in the ED. Subsequent CT imaging, however, suggested findings more consistent with infection or inflammation rather than neoplastic disease. While AI testing in this case would have allowed definitive diagnosis of Lyme neuroborreliosis, CSF antibodies at the time of lumbar puncture were unfortunately not obtained. Ultimately, the high likelihood of Lyme neuroborreliosis as the cause of this patient's SIADH was supported by positive serum Lyme-specific testing, the ruling out of other infectious causes of meningitis, and resolution of neurologic symptoms and hyponatremia with appropriate antibiotic treatment.

## Conclusions

Neurologic manifestations of Lyme disease are somewhat common but highly variable. As exemplified in this case, these broad manifestations can delay proper management, especially in the absence of hallmark Lyme-associated symptoms, such as erythema migrans or a flu-like prodrome. This case is notable for its combination of refractory hyponatremia and a neurologic picture resembling demyelinating conditions, highlighting the diagnostic complexity of Lyme neuroborreliosis. Key takeaways include the importance of considering Lyme neuroborreliosis in atypical neurologic presentations that resemble other demyelinating pathologies, such as multiple sclerosis. Additionally, this case further contributes to the growing body of literature linking Lyme neuroborreliosis to SIADH, a relationship that remains underreported but clinically significant.
